# Cardiovascular Risk Factors Associated With the Metabolically Healthy Obese (MHO) Phenotype Compared to the Metabolically Unhealthy Obese (MUO) Phenotype in Children

**DOI:** 10.3389/fendo.2020.00027

**Published:** 2020-02-07

**Authors:** Simonetta Genovesi, Laura Antolini, Antonina Orlando, Luisa Gilardini, Simona Bertoli, Marco Giussani, Cecilia Invitti, Elisa Nava, Maria Grazia Battaglino, Alessandro Leone, Maria Grazia Valsecchi, Gianfranco Parati

**Affiliations:** ^1^School of Medicine and Surgery, University of Milano-Bicocca, Milan, Italy; ^2^Cardiologic Unit, Istituto Auxologico Italiano, IRCCS, Milan, Italy; ^3^Center of Biostatistics for Clinical Epidemiology, School of Medicine and Surgery, University of Milano-Bicocca, Monza, Italy; ^4^International Center for the Assessment of Nutritional Status (ICANS), Department of Food, Environmental and Nutritional Sciences (DeFENS), University of Milan, Milan, Italy; ^5^Lab of Nutrition and Obesity Research, Istituto Auxologico Italiano, IRCCS, Milan, Italy; ^6^Family Pediatrician, ATS Milan, Milan, Italy; ^7^Nephrology and Dialysis Unit, IRCCS Multimedica, Sesto San Giovann, Italy

**Keywords:** children, metabolically healthy obesity, Uric Acid, HOMA index, waist-height ratio

## Abstract

**Background:** In pediatric age the prevalence of obesity is high. Obese children who do not have other risk factors than excess weight have been defined as “metabolically healthy obese” (MHO).

**Aim:** The aim of this study is to evaluate, in a population of obese children, the prevalence of the MHO and “metabolically unhealthy obese” (MUO) phenotype. Furthermore, we evaluated the distribution of Uric Acid, HOMA index and Waist-Height ratio (W-Hr) in the MHO and MUO sub-groups and the impact of these non-traditional risk factors on the probability to be MUO.

**Methods:** In 1201 obese children and adolescents [54% males, age (±SD) 11.9 (±3.0) years] weight, height, waist circumference, systolic (SBP) and diastolic (DBP) blood pressure, pubertal status, glucose, insulin, HDL cholesterol, triglycerides and Uric Acid serum values were assessed. MUO phenotype was defined as the presence of at least one of the following risk factors: SBP or DBP ≥ 90th percentile, glycaemia ≥ 100 mg/dl, HDL cholesterol <40 mg/dl, triglycerides ≥100 mg/dl (children <10 years) or ≥130 mg/dl (children ≥10 years). A multivariate logistic regression analysis was used to estimate the association between MUO phenotype and non-traditional cardiovascular risk factors.

**Results:** The prevalence of the MUO status was high (61%). MUO subjects were more often male, older and pubertal (*p* < 0.001). The levels of the three non-traditional risk factors were significantly higher in MUO children compared to MHO children (*p* < 0.001) and all of them were independent predictors of the fact of being MUO [OR 1.41 (95% CI 1.24–1.69); 1.15 (95% CI 1.06–1.23) and 1.03 (95% CI1.01–1.05) for Uric Acid, HOMA index and W-Hr, respectively]. About 15% of MHO subjects had serum Uric Acid, HOMA index and W-Hr values within the highest quartile of the study population.

**Conclusion:** The prevalence of MUO subjects in a large pediatric population is high and serum Uric Acid, HOMA index and W-Hr values are independent predictors of the probability of being MUO. A non-negligible percentage of subjects MHO has high values of all three non-traditional risk factors.

## Introduction

The increasing prevalence of obesity in children and adolescents is becoming a global concern (1). Obesity is a complex, chronic condition characterized by multiple causes and adverse health consequences (2, 3) and it is associated with increase all-cause mortality in adults (4). Excess weight in childhood is a major risk factor for adulthood obesity (5) and increases the risk of morbidity and mortality later in life (6). Not all obese individuals show an equal health risk. Metabolically healthy obesity (MHO) is defined as a condition in which, despite the significant excess weight, traditional risk factors as insulin resistance (IR), dyslipidemia, and hypertension are not present (7–10), contrary to what occurs in the metabolically unhealthy obesity (MUO) condition.

The MHO phenotype has been described since the 1980s (11). For adults MHO is generally defined as a condition in which metabolic syndrome (MetS) factors are absent (2, 12).

For children and adolescents, there is still no universally accepted definition of MetS (13, 14). For this reason, the American Academy of Pediatrics recommended to focus on the concept of cardiometabolic risk factor clustering, rather than on the presence of MetS to define MHO in pediatric age (15). Recently, a consensus of international experts proposed a definition of MHO including cutoff values for high density lipoprotein-cholesterol [>40 mg/dl, triglycerides ≤ 150 mg/dl), systolic and diastolic blood pressure (≤ 90th percentile), and a measure of glycaemia (16).

In adulthood, individuals with MUO show increased mortality rates and cardiovascular risk compared to obese individuals with normal metabolic profiles (17). It is therefore possible that children and adolescents with the MUO phenotype may have higher cardiovascular and mortality risk compared to MHO subjects when they become adults.

The aims of the present study are (i) to determine the prevalence of the MUO phenotype in a population sample of obese Italian children and adolescents referred to secondary level Clinical Centers, (ii) to evaluate the distribution of Waist-Height ratio (W-Hr), Homeostatic Model Assessment (HOMA) index of insulin resistance and serum Uric Acid values in MHO and MUO children (iii) to investigate whether these non-traditional cardiovascular risk factors are independent predictors of the MUO phenotype, if added to the risk factors already present in the definition.

## Methods

We studied a cohort of 1201 obese children and adolescents (4–18 years) referred from January 2002 to August 2019 by their primary care pediatricians to the IRCCS Istituto Auxologico Italiano and to the International Center for the Assessment of Nutritional Status, ICANS. Cardiovascular risk factors were evaluated at Istituto Auxologico Italiano, IRCCS, Cardiologic Unit. Inclusion criteria for the study were: (1) age ≤18 years (2) informed consent obtained from parents or legal representatives to participate in the study. Exclusion criteria were: (1) syndromic obesity; (2) dysthyroidism; (3) diabetes; (4) congenital cardiovascular diseases (5) any form of secondary hypertension (6) presence of chronic kidney disease and (7) treatment with antihypertensive drugs, lipid-lowering drugs, and glucose altering medications such as metformin.

The study protocol was approved by the local institutional ethics committee and conformed to the ethical guidelines of the 1975 Declaration of Helsinki.

### Anthropometric Parameters and Blood Pressure

Anthropometric measurements were taken following international guidelines (18). Weight was measured with 700 SECA gram scale and height with 417 SECA stadiometer (®SECA Medical Measuring Systems and scales, Birmingham UK), approximated to the nearest 100 g and 0.5 cm, respectively. BMI was calculated as weight (kg)/height (m)^2^. BMI z-scores were calculated using the Centers for Disease and Control prevention charts available at http://www.cdc.gov/nchs/. Weight class (Obesity) was defined according to the International Obesity Task Force classification (19). Waist circumference was measured in standing position at the midpoint between the last rib and the iliac crest with a non-stretch tape to the nearest 0.5 cm. Waist–height ratio was calculated dividing WC by height obtaining a pure number. Pubertal stage was assessed by a medical examination and children were classified into two categories: pre-pubertal and pubertal according to Tanner (20). Blood pressure (BP) was measured using an aneroid sphygmomanometer with the appropriate cuff for the child's upper arm size (Heine GAMMA® G7, Germany). The sphygmomanometer was calibrated before starting the study and once a month thereafter with a mercury sphygmomanometer. Systolic BP (SBP) was defined by the first Korotkoff sound (appearance of sounds) and diastolic BP (DBP) was identified by the fifth Korotkoff sound (disappearance of sounds). Measurements were performed after at least 5 min of rest. BP measures were taken 3 times (at 3–5 min intervals). The average of the two last SBP and diastolic BP (DBP measurements was calculated. Systolic BP and DBP 90th percentiles were calculated according to the nomograms of the National High Blood Pressure Education Program (NHBPEP) Working Group on High Blood Pressure in Children and Adolescents (21).

### Biochemical Parameters

Fasting blood samples were drawn after a 12-h fasting period in order and analyzed in the same morning. Glucose, triglycerides, HDL- cholesterol, alanine transaminase (ALT), gamma- glutamyl-transferase (GGT) and uric acid were measured by means of an enzymatic method (Cobas Integra 400 Plus, Roche Diagnostics, Rotkreuz, Switzerland), with intra-and inter-assay CVs < 2%. Circulating insulin was measured in duplicate by an autoanalyzer (Cobas e411 Hitachi, Roche Diagnostics). The homeostatic model assessment-insulin resistance was calculated as [fasting glucose (mg/dL) × fasting insulin (mU/L)/405] (22).

#### MHO/MUO Definition

The following definition to identify MHO subjects was applied: SBP and DBP <90^th^ percentile by gender, age and height percentile, glycaemia <100 mg/dl, HDL cholesterol >40 mg/dl, triglycerides <100 mg/dl (children <10 years) or <130 mg/dl (children ≥10 years) (16).

MUO phenotype was defined as the presence of at least one of the following risk factors: SBP or DBP≥ 90th percentile, glycaemia >100 mg/dl, HDL cholesterol ≤40 mg/dl, triglycerides ≥100 mg/dl (children <10 years) or ≥130 mg/dl (children ≥10 years).

In the proposed definition of MHO (16) it is not specified which glycemia cut-off should be used. However, most of the studies reviewed by the expert consensus used fasting glucose <100 mg/dL. For this reason, we used the same value in our study. The triglyceride cut-offs were based on the indications of the National Cholesterol Education Program (NCEP) Expert Panel on Cholesterol Levels in Children (23, 24). Since the proposed definition of MHO suggests 150 mg as a single cut-off for triglycerides, we also performed a further analysis (sensitivity analysis) by entering this triglyceride cut-off.

### Statistical Method

Data analysis was conducted separately for male and female subjects. Categorical variables were described by percentages and continuous variables were reported as means and standard deviations. Comparison across groups was performed by Chi-square and *T*-test considering 0.05 as significance level to judge *p*-values. Logistic regression was used to relate the binary status (MHO or MUO) to explanatory variables through univariate and multivariate models. The discrimination potential of explanatory variables was investigated via receiver operating characteristic curves. Data analysis and graphics was conducted by the STATA 16 software (StataCorp LLC 4905 Lakeway Drive College Station, TX 77845 USA).

## Results

Table 1 shows the characteristics of the study population by gender. About 46.0% of the subjects were male and 56% were pubertal. Serum Uric Acid and W-Hr values were significantly higher in males [5.1 (±1.5) vs. 4.7 (±1.0) mg/dl, *p* < 0.001 and 60.6 (±8.4) vs. 59.4 (±7.4)%, *p* = 0.016, respectively].

**Table 1:** Characteristics of the study population by gender.

**Variable**	**Male** **(*n* = 559)**	**Female** **(*n* = 642)**	**Total**	***p*-value**
Age years (±SD)	12.0 (±3.2)	11.8 (±2.7)	11.9 (±3.0)	0.135
Puberty (yes,%)	257 (46.0%)	420 (65.4%)	677 (56.4%)	<0.001
BMI, kg/m^2^ (±SD)	32.0 (±4.6)	28.0 (±5.3)	30.5 (±5.3)	<0.001
BMI z-score (±SD)	2.2 (±0.3)	2.3 (±0.3)	2.2 (±0.3)	<0.001
SBP, mmHg (±SD)	111 (±13.8)	116 (±12.7)	113 ± (13.4)	<0.0001
SBP z-score (±SD)	0.68 (±1.23)	0.40 (±1.18)	0.53 (±1.21)	<0.0001
DBP, mmHg (±SD)	71 (±8.2)	70 (±8.1)	70.3 (±8.2)	0.030
DBP z-score (±SD)	0.63 (±0.72)	0.59 (±0.69)	0.61 (±0.70)	<0.0001
Glycaemia, mg/dl (±SD)	85.1 (±7.5)	83.5 (±7.3)	84.2 (±7.4)	<0.001
Triglycerides, mg/dl (±SD)	81.6 (±41.6)	82.6 (±39.3)	82.1 (±40.4)	0.699
HDL cholesterol, mg/dl (±SD)	49.7 (±11.1)	49.0 (±10.5)	49.3 ± (10.8)	0.856
W-Hr,%, (±SD)	60.6 (±8.4)	59.4 (±7.4)	60.0 (±7.9)	0.016
Uric Acid, mg/dl) (±SD), *n* = 1,082	5.1 (±1.5)	4.7 (±1.1)	4.9 (±1.3)	<0.001
HOMA index (±SD), *n* = 1,142	3.3 (±2.2)	3.3(±2.3)	3.3 (±2.3)	0.813

As expected, the pre-pubertal subjects were younger, more sensitive to insulin, and had lower Uric Acid and W-Hr values than pubertal, [age, 9.2 (±1.9) vs. 13.7 (±2.3) years, *p* < 0.001; HOMA index 2.8 (±1.9) vs. 3.7 (±2.4), *p* < 0.001; Uric Acid 4.4 (±0.9) vs. 5.2 (±1.3) mg/dl, *p* < 0.001 and W-Hr58.0 (±7.0) vs. 61.0 (±8.0)%, *p* < 0.001].

Seven hundred and thirty-two subjects (61.0%) were MUO. Table 2 shows the characteristics of the study population by metabolic status. MUO subjects were more frequently male [OR 1.26 (95% CI 0.99–1.59), *p* = 0.053], and more often pubertal [OR 2.0 (95% CI 1.56–2.55), *p* < 0.001] compared to MHO children. The risk of being MUO increased with increasing age [OR 1.14 (95% CI 1.09–1.18), per 1-year increment, *p* < 0.001]. Both SBP and DBP z-scores were higher in MUO compared to MHO subjects (*p* < 0.001). Moreover, plasma values of glycaemia and triglycerides were higher and those of HDL cholesterol were lower in MUO children (*p* < 0.001). Serum Uric Acid values were significantly higher in the MUO group than in the MHO group [5.2 (±1.3) vs. 4.5 (±1.1) mg/dl, *p* < 0.001], as well as those of HOMA index [3.7 (±2.4) vs. 2.8 (±1.8), *p* < 0.001] and W-Hr [61.2 (±8.1) vs. 58.0 (±7.2)%, *p* < 0.001]. It is interesting to note that MUO subjects were not different from MHO subjects as far as the BMI z-score values were concerned (*p* = 0.117).

**Table 2:** Characteristics of the study population by metabolic status.

**Variable**	**MHO** **(*n* = 469)**	**MUO** **(*n* = 732)**	***p*-value**
Male (%)	202 (36.1)	357 (63.9)	0.053
Female (%)	267 (41.6)	375 (58.4)	
Age years (±SD)	11.2 (±3.0)	12.3 (±3.0)	<0.001
Puberty (yes,%)	222 (47.3)	455 (62.2)	<0.001
BMI, kg/m^2^ (±SD)	30.0 (±5.7)	30.7 (±5.0)	0.025
BMI z-score (±SD)	2.3 (±0.3)	2.2 (±0.3)	0.117
SBP, mmHg (±SD)	105.6 (±9.4)	118.3 (±13.3)	<0.001
SBP z-score (±SD)	−0.12 (±0.89)	0.95 (±1.21)	<0.001
DBP, mmHg (±SD)	66.9 (±6.7)	72.5 (±8.3)	<0.001
DBP z-score (±SD)	0.32 (±0.54)	0.80 (±0.73)	<0.001
Glycaemia, mg/dl (±SD)	83.2 (±6.7)	84.9 (±7.8)	<0.001
Triglycerides, mg/dl (±SD)	65.0 (±20.8)	96.0 (±46.6)	<0.001
HDL cholesterol, mg/dl (±SD)	53.4 (±9.2)	46.7 (±11.0)	<0.001
W-Hr, % (±SD), *n* = 1,138	58.0 (±7.2)	61.2 (±8.1)	<0.001
Uric Acid, mg/dl (±SD), *n* = 1,082	4.5 (±1.1)	5.2 (±1.3)	<0.001
HOMA index (±SD), *n* = 1,142	2.8 (±1.8)	3.7 (±2.4)	<0.001

Among MUO children, 389 (53.1%) presented only one risk factor, 266 (36.3%) 2 risk factors, 57 (7.8%) 3 risk factors and only 20 (2.7%) had all four risk factors of the definition of the MUO phenotype. Table 3 shows the risk factor distribution (SBP ≥ 90th percentile, DBP ≥ 90th percentile, low HDL cholesterol, high triglycerides and high glycaemia) among MUO children according to gender. Hypertriglyceridemia and high SBP were the most represented risk factor (42.6%), subjects with DBP≥ 90^th^ percentile and those with low HDL were also frequent (33.2%). Instead, a low prevalence of elevated glycaemia was observed (8.5%).

**Table 3:** Distribution of risk factors among MUO children by gender.

	**Females** **(*n* = 375)**	**Males** **(*n* = 357)**	**Total** **(*n* = 732)**
Systolic blood pressure ≥90th percentile *n* (%)	138 (36.8%)	174 (48.7%)	312 (42.6%)
Diastolic blood pressure ≥90th percentile *n* (%)	118 (31.5%)	125 (35.0%)	243 (33.2%)
HDL cholesterol <40 mg/dl *n* (%)	125 (33.3%)	118 (33.0%)	243 (33.2%)
Triglycerides ≥100/130 mg/dl *n* (%)	168 (44.8%)	144 (40.3%)	312 (42.6%)
Glycaemia ≥100 mg/dl *n* (%)	29 (7.7%)	33 (9.2%)	62 (8.5%)

After adjustment for gender, age and pubertal status, Uric Acid values [OR 1.53 (95% CI 1.35–1.74), *p* < 0.001], those of HOMA index [OR 1.28 (95% CI 1.12–1.30), *p* < 0.001] and those of W-Hr [OR 102.23 (95% CI 15.80–661.51), *p* < 0.001] were all independent predictors of the probability of being MUO.

When Uric Acid, HOMA index and W-Hr were put together in the logistic regression model, all three variables maintained their predictive power on the probability of being MUO and the result did not change after adjustment for gender, age and pubertal status. The probability of being classified as MUO increased by 41% for each 1 mg/dl increase in serum Uric Acid, by 15% for each increment of 1 unit of HOMA index and by 3% for each percentage unit of W-Hr (Table 4).

**Table 4:** Effect of Uric Acid, HOMA index and W-Hr on the risk to be MUO by a multiple logistic regression.

**Variable**	**Odds ratio**	**95% Confidence interval**	***p*-value**
**UNIVARIATE ANALYSIS**			
Uric Acid (mg/dl)	1.48	(1.30–1.67)	<0.001
HOMA index	1.15	(1.07–1.24)	<0.001
W-Hr (%)	1.03	(1.01–1.05)	0.001
**MULTIVARIATE ANALYSIS**			
Gender (male)	1.26	(0.95–1.68)	0.110
Puberty (yes vs. no)	1.32	(0.89–1.95)	0.161
Age (years)	1.01	(0.95–1.08)	0.614
Uric Acid (mg/dl)	1.41	(1.24–1.62)	<0.001
HOMA index	1.15	(1.06–1.23)	<0.001
W-Hr (%)	1.03	(1.01–1.05)	0.006

**Figure 1:**
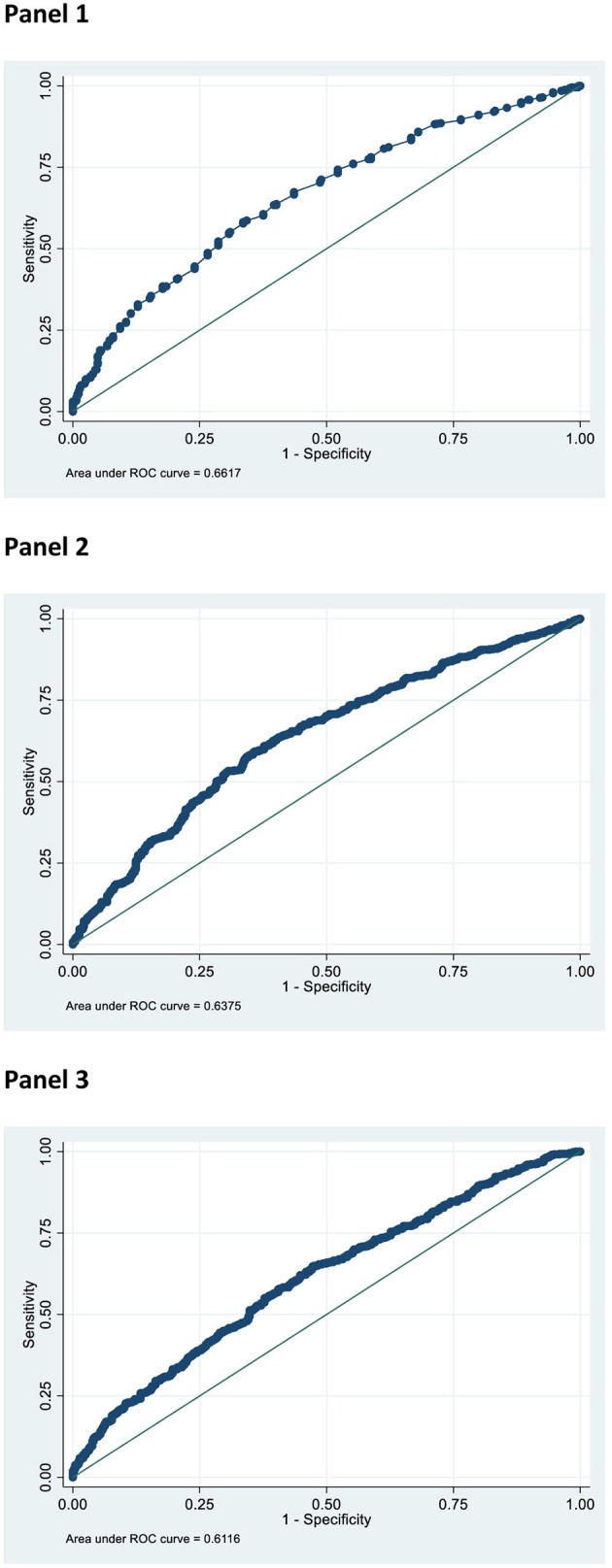
Receiver Operating Characteristic (ROC) curve of Uric Acid (mg/dl) **(Panel 1)**, HOMA index **(Panel 2)**, and W-Hr **(Panel 3)** to detect the condition of metabolic unhealthy obesity.

Figure 1 shows the three ROC curves related to the sensitivity and specificity of Uric Acid (Panel 1), HOMA index (Panel 2) and W-Hr (Panel 3) in the prediction of being a MUO subject. The areas under ROC curves were 0.66, 0.64, and 0.61 for Uric Acid, HOMA index and W-Hr, respectively.

A high percentage of MUO children showed Uric Acid, HOMA index and W-Hr values within the highest quartile. In the MUO group the percentage of children who had Uric Acid, HOMA index and W-Hr values within the fourth quartile was about twice the percentage observed in the MHO group (*p* < 0.001) (Table 5).

**Table 5:** Children with Uric Acid, HOMA index and W-Hrvalues within the fourth quartile in MHO/MUO groups.

**Variable**	**4th quartile** ***n*** (%^*^)		***p*-value**
	**MHO *n* = 496**	**MUO *n* = 732**	
Uric Acid (mg/dl)	65 (15.1)	227 (34.8)	<0.001
HOMA index	70 (15.2)	216 (31.6)	<0.001
W-Hr (%)	76 (17.2)	210 (30.2)	<0.001

* *Percentages are calculated on the total number of subjects where the variable was assessed*.

The sensitivity analysis performed by entering 150 mg/dl as single triglyceride cut-off confirmed the independent predictive value of Uric Acid, HOMA index and W-Hr on the risk of being MUO. With the additional analysis, BMI z-score was greater in MHO compared to MUO subjects, however the difference disappeared at multivariate analysis. Furthermore, male gender was an independent predictor of MUO phenotype (Supplementary Tables 2–5). The areas under ROC curves did not change with the sensitivity analysis.

## Discussion

The main results of our study are: (i) the prevalence of MUO subjects in a large pediatric population is high (about 60%) (ii) three risk factors not included in the definition of MHO (serum Uric Acid, HOMA index and W-Hr) are significantly higher in MUO children compared to MHO children and all three are independent predictors of the possibility of being MUO iii) considering the study population, a non-negligible percentage of MHO subjects has values of serum Uric Acid, HOMA index and W-Hr within the highest quartile.

The prevalence of the MHO phenotype in children described in the literature varies from 20 to 68%, but it should be emphasized that the MHO definitions used in the different studies are not always the same. Moreover, in most cases these studies are performed in populations composed of a relatively small number of children (2). The prevalence of MHO subjects we found in a large sample is higher than that described by other authors (25). However, it must be considered that this sample does not originate from a population screening. These young patients, in fact, are referred to us by their family pediatricians, when they have some difficulty in managing them. Our clinics, in fact, constitute a second-level clinical care center. For this reason, we cannot consider the study population as representative of the Italian pediatric population. The prevalence of individual risk factors in our sample of MUO children is similar to that reported in a previous study performed in a large pediatric population (26).

Previous studies demonstrated that all three non-traditional cardiovascular risk factors considered have a significant and positive association with BP z-scores and the presence of arterial hypertension in pediatric populations (27–31). The well-documented association of hyperuricemia with metabolic syndrome in adults (32) agrees with the observation made in a cohort of children with severe obesity (33), and further supports the role for Uric Acid as an independent risk factor for cardiovascular diseases. Several studies show that MUO subjects have a higher degree of obesity (i.e., a BMI z-score) than MHO subjects (34, 35). In our population, on the contrary, there is no difference in BMI z-score values between MHO children and MUO children. Despite this, W-Hr is significantly higher in MUO subjects, suggesting the presence of a different distribution of abdominal adipose tissue in this sub-group. In children, a high W-Hr value is associated with alterations of a series of cytokines that can lead to negative consequences on the cardiovascular system (36–38). In the present study population, although there are no significant differences in BMI z-score between the two phenotypes, the values of HOMA index are significantly higher in MUO children. This finding suggests a close relationship between visceral fat and insulin resistance, a known cardiovascular risk factor (29). The INTERHEART study, a case-control study that looked at 29,972 patients in 52 countries, showed that the relationship between waist circumference and waist-to-hip ratio and myocardial infarction is stronger than the relationship between myocardial infarction and BMI (39).

The abovementioned evidence helps us to interpret our results, suggesting a series of reasons why, individually, the non-traditional cardiovascular risk factors considered are associated with the MUO phenotype. However, our study provides an additional observation concerning these data. As a matter of fact, in our population serum Uric Acid, HOMA index and W-Hr may be helpful, independently of each other, to identify among obese children those showing the MUO phenotype. It is important to underline that 15% of children with the MHO phenotype show high values of one or more of the three non-traditional cardiovascular risk factors. It has been shown that obesity is a powerful predictor of cardiac hypertrophy in children, regardless of the presence of hypertension (40). Di Bonito et al. described a prevalence of hepatic steatosis of 36.5% and a proportion of 56.0% of left ventricular hypertrophy in a pediatric MHO population (41). Moreover, Dangardt et al. showed, in a large population of children and adolescents, that persistence of high total fat mass during adolescence, assessed using dual-energy x-ray absorptiometry, was positively associated with increased arterial stiffness, assessed by carotid-femoral pulse wave velocity. This adverse association (with arterial stiffness) was absent with BMI z-score (42). It is therefore conceivable that even MHO subjects cannot be considered truly “healthy” and that they may present initial organ damage of some kind or that they may, over time, become hypertensive.

The definition of an MHO phenotype was created in the adult population, in which it is possible to evaluate the incidence of cardiovascular events and mortality, and to compare this between MHO and MUO patients (17). Of course, this cannot be done in the pediatric population. Moreover, in adults, obesity is frequently a stable condition, not of recent onset, and may therefore exert its negative effects over time. In children, instead, the presence of obesity can be transient and represents a condition with a more recent clinical history than in adults. However, Reinehret al. demonstrated that, in their large pediatric population, after 1 year of follow-up, the majority of the MHO children remained MHO (68.0%) (26). Our data show that older and post-pubertal subjects, in whom it can be assumed that obesity has been present for longer, are more frequently MUO. This would suggest that it takes some time for metabolic alterations to occur. Furthermore, for what regards non-traditional risk factors, it should be noted that also insulin resistance and Uric Acid serum values increase with age and puberty. Our multivariate model however, shows an association between MHO phenotype and these non-traditional risk factors independent of the age of the child, suggesting the importance of a very early intervention. It is reasonable to hypothesize that a part of the children who present the MHO phenotype could become MUO adults; this could be influenced by their genetic predisposition, as well as by eating habits and lifestyle (43). Only prospective studies will allow us to understand what percentage of children who are obese in childhood remain obese in adulthood and how many of the subjects showing the MHO phenotype in childhood, continue to be MHO even when they become adults.

A strength of the study is the size of the study-population, but a limitation is the cross-sectional design. For this reason, we can only describe associations between cardiovascular risk factors and the presence of MU phenotype, but we cannot say whether or not there is a cause/effect relationship. In addition, our sample is made up of subjects referred to a Center for the prevention and treatment of cardiovascular risk in pediatric age and may not represent the totality of obese children.

In conclusion, on the one hand distinguishing between the MHO and MUO phenotype in children can be potentially useful, as it allows the pediatrician to more closely follow-up the subjects with highest cardiovascular risk (8). The definition proposed by Damanhoury et al. (16) however, can lead to an underestimation of the number of obese children actually at risk, as obesity *per se* can lead to early organ damage even in the absence of metabolic alterations (40, 41). Furthermore, the definition does not cover all potential cardiovascular risk factors, such as insulin resistance, high levels of Uric Acid and visceral adiposity (44). In our opinion, the definition proposed by Damanhoury et al. (16) is a bit limiting and pediatricians would need a more detailed definition to identify obese MUO subjects.

## Data Availability Statement

The datasets generated for this study are available on request to the corresponding author.

## Ethics Statement

The studies involving human participants were reviewed and approved by Ethics committee of Istituto Auxologico Italiano, RICARPE Study, code 2015_10_20_02. Written informed consent to participate in this study was provided by the participants' legal guardian/next of kin.

## Author Contributions

SG and MG conceptualized and designed the study, drafted the initial manuscript, and reviewed and revised the manuscript. AO, EN, MB, MG, and AL collected data. LA and MV performed data analysis. LG, SB, CI, and GP reviewed and revised the manuscript. All authors approved the final manuscript as submitted and agree to be accountable for all aspects of the work.

### Conflict of Interest

The authors declare that the research was conducted in the absence of any commercial or financial relationships that could be construed as a potential conflict of interest. The handling Editor declared a past co-authorship with the authors LG and CI.
