# Supplementary material for: Cardiovascular Risk Factors Associated With the Metabolically Healthy Obese (MHO) Phenotype Compared to the Metabolically Unhealthy Obese (MUO) Phenotype in Children
Source: Front Endocrinol (Lausanne). 2020 Feb 7;11:27. doi: 10.3389/fendo.2020.00027 (PMC7025459; doi:10.3389/fendo.2020.00027)
Supplement: Supplementary file 1 [file Table_1.docx]

**TableS 1:** Characteristics of the study population by metabolic status.

| **Variable** | **MHO (n=506)** | **MUO (n=695)** | **p value** |
| --- | --- | --- | --- |
| **Male (%)** | 214 (42.3) | 345 (49.6) | 0.012 |
| **Female (%)** | 292 (57.7) | 350 (50.4) |  |
| **Age years (±SD)** | 11.1 (**±**2.9) | 12.5 (**±**2.8) | <0.001 |
| **Puberty (yes,%)** | 235 (46.4) | 442 (63.6) | <0.001 |
| **BMI, kg/m^2^ (±SD)** | 30.1 (**±**5.7) | 30.8 (**±**4.9) | 0.012 |
| **BMI z-score (±SD)** | 2.3 (**±**0.3) | 2.2 (**±**0.3) | 0.0162 |
| **SBP, mmHg (±SD)** | 105.6 (**±**9.3) | 119.0 (**±**13.1) | <0.001 |
| **SBP z-score (±SD)** | -0.11 (**±**0.88) | 1.01 (**±**1.20) | <0.001 |
| **DBP, mmHg (±SD)** | 66.8 (**±**6.5) | 72.9(**±**8.3) | <0.001 |
| **DBP z-score (±SD)** | 0.32 (**±**0.53) | 0.82 (**±**0.74) | <0.001 |
| **Glycaemia, mg/dl (±SD)** | 83.1 (**±**6.6) | 85.1 (**±**7.9) | <0.001 |
| **Triglycerides, mg/dl (±SD)** | 69.3 (**±**25.6) | 94.1 (**±**47.4) | <0.001 |
| **HDL cholesterol, mg/dl (SD)** | 53.1 (**±**9.1) | 46.5 (**±**11.2) | <0.001 |
| **W-Hr, % (±SD), n=1138** | 58.0 (**±**7.3) | 61.4 (**±**8.3) | <0.001 |
| **Uric Acid, mg/dl (±SD), n=1082** | 4.5 (**±**1.1) | 5.2 (**±**1.3) | <0.001 |
| **HOMA index (±SD), n=1142** | 2.8 (**±**1.8) | 3.7 (**±**2.5) | <0.001 |

**TableS 2:** Distribution of risk factors among MUO children by gender

|  | **Females**  **(n=350)** | **Males**  **(n=345)** | **Total**  **(n=695)** |
| --- | --- | --- | --- |
| **Systolic blood pressure >90^th^ percentile n (%)** | 138 (39.4%) | 174 (50.4%) | 312 (44.9%) |
| **Diastolic blood pressure >90^th^ percentile n (%)** | 118 (33.7%) | 125 (36.2%) | 243 (35.0%) |
| **HDL cholesterol *<*40 mg/dl n (%)** | 125 (35.7%) | 118 (34.2%) | 243 (35.0%) |
| **Triglycerides <100/130 mg/dl n (%)** | 121 (34.6%) | 103 (29.9%) | 224 (32.2%) |
| **Glycaemia >100 mg/dl n (%)** | 29 (8.3%) | 33 (9.6%) | 62 (8.9%) |

**TableS 3:** Effect of Uric Acid, HOMA index and W-Hr on the risk to be MUO by a multiple logistic regression.

| **Univariate**  **Analysis** | **Variable** | **Odds Ratio** | **95% Confidence Interval** | **p value** |
| --- | --- | --- | --- | --- |
|  | **Uric Acid (mg/dl)** | 1.48 | (1.31-1.67) | < 0.001 |
|  | **HOMA index** | 1.13 | (1.06-1.21) | 0.001 |
|  | **W-Hr (%)** | 1.04 | (1.02-1.06) | <0.001 |
| **Multivariate**  **Analysis** | **Variable** | **Odds Ratio** | **95% Confidence Interval** | **p value** |
|  | **Gender (male)** | 1.44 | (1.07-1.92) | 0.014 |
|  | **Puberty (yes vs no)** | 1.37 | (0.93-2.02) | 0.117 |
|  | **Age (years)** | 1.03 | (0.96-1.10) | 0.448 |
|  | **Uric Acid (mg/dl)** | 1.40 | (1.23-1.60) | <0.001 |
|  | **HOMA index** | 1.13 | (1.05-1.21) | 0.001 |
|  | **W-Hr (%)** | 1.04 | (1.01-1.06) | 0.001 |
|  | **BMI z score** | 0.67 | (0.42-1.08) | 0.103 |

**TableS 4:** Children with Uric Acid, HOMA index and W-Hr values within the fourth quartile in MHO and MUO groups.

| **Variable** | **4th quartile n (%*)** | | **p value** |
| --- | --- | --- | --- |
|  | MHO n=506 | MUO n=695 |  |
| **Uric Acid (mg/dl)** | 82 (16.6) | 204 (31.5) | <0.001 |
| **HOMA index** | 70 (15.2) | 222 (35.7) | <0.001 |
| **W-Hr (%)** | 84 (17.7) | 202 (30.5) | <0.001 |

***=percentages are calculated on the total number of subjects where the variable was assessed**
